# In-Hospital Outcomes of Female Patients With Inferior Wall Myocardial Infarction

**DOI:** 10.7759/cureus.13274

**Published:** 2021-02-11

**Authors:** Ghulam Kubra, Tahir Saghir, Shazia Rasheed, Fariha Hasan Rehan, Asad Ali, Syed Abbas

**Affiliations:** 1 Electrophysiology, National Institute of Cardiovascular Diseases, Karachi, PAK; 2 Cardiology, National Institute of Cardiovascular Diseases, Karachi, PAK; 3 Echocardiography, National Institute of Cardiovascular Diseases, Karachi, PAK

**Keywords:** inferior wall mi, female, outcome, complete heart block

## Abstract

Background

The aim of this study was to determine the in-hospital outcome of female patients with inferior wall myocardial infarction (MI).

Methodology

This study was conducted from January to December 2017 at the Department of Cardiology, National Institute of Cardiovascular Disease, Karachi. A total of 59 women admitted with inferior wall MI were enrolled in the study. In all patients, in-hospital outcomes were observed. Descriptive statistics were applied. Stratification was done using chi-square test, and p-value of ≤0.05 was considered significant.

Results

The mean age of study participants was 58.80 ± 9.17 years, while 247 (79.7%) participants were above 50 years of age. The mean onset of duration of sign and symptoms of inferior wall MI was 3.48 ± 1.53 hours. There were 36 (61.0%) patients who had diabetes mellitus, 46 (78.0%) had hypertension, 17 (28.8%) were obese, nine (15.3%) had a family history of MI, and three (5.1%) were smokers. There were 43 (72.9%) patients who were illiterate. In our study, eight (13.6%) females were found to have sinus bradycardia, seven (11.9%) had sinus tachycardia, three (5.1%) had atrial fibrillation, and 24 (40.7%) had complete heart block. Mortality was noted in five (8.5%) patients.

Conclusions

Women with an acute inferior wall MI had a higher rate of complete heart block and adverse in-hospital outcomes. Female gender itself with inferior wall MI may be at risk for in-hospital adverse outcomes.

## Introduction

Ischemic heart disease (IHD) is known to have a wide clinical spectrum that ranges from stable angina through unstable angina to myocardial infarction (MI) [1]. IHD has been found to be more prevalent among male patients [2]. Common risk factors for IHD are diabetes, hypertension, smoking, hyperlipidemia, and family history of IHD. MI is the necrosis of myocytes because of disruption in the blood supply [2,3]. MI can be anterior, inferior, lateral, or posterior walls of left ventricle depending upon the vessel supplying the area [4].

Inferior wall MI has been found to account for 40-50% of acute MI cases; however, they have better favorable outcomes compared to other types of MI, for example, mortality ranges between 2% and 9% in these cases [5]. Around 50% of patients having inferior wall MIs are thought to have complications that significantly amend favorable prognosis [6]. Patient with inferior wall MIs having precordial ST segment depression, complete atrioventricular block (AVB), or right ventricular infarction (RVI) are high-risk group patients as they have a larger area at risk [7,8]. Inferior wall MI and heart block are thought to result in increased incidence of early mortality. Multi-vessel disease and high-grade blockage of the left anterior descending coronary artery have been found in about 90% of the cases developing complete AV blockage after inferior wall MI [9]. In comparison to anterior wall MIs, bradyarrhythmias occur more frequently among patients of inferior wall MIs. Sinus bradycardias are the most prevalent type of bradyarrhythmias seen in four to six hours among patients of acute MIs [10]. AVB occurs in 9-33% of inferior wall MI cases, while blockage or early onset generally lasts for a short duration [11].

A study from Iran concluded that rates of heart failure and in-hospital deaths increased among female gender [12]. According to the American Heart Association, mortality burden has been found to be more among females since 1984 [13]. Gender differences occur in terms of pathophysiology as well as clinical presentation of MIs and have been found to result in treatment delays [13]. The current study aimed to determine the in-hospital outcomes of female patients with inferior wall MI.

## Materials and methods

This study was conducted from January to December 2017 at the Department of Cardiology, National Institute of Cardiovascular Disease, Karachi. Approval from institutional ethical review board was acquired. Written consent was taken from all enrolled patients or from their guardians.

A total of 59 female patients aged 35-80 years with inferior wall MI and presenting within three days of having symptoms of inferior wall MI were enrolled. The diagnosis of inferior wall MI was labeled as the presence of ST segment elevation in leads I, II, and aVF; Q waves more than >0.04 seconds in duration in lead I, II, and aVF; or reciprocal ST segment depression in the lateral and/or high lateral leads (I, aVL, V5, and V6) and at least one of the following: (1) chest pain considered characteristic of myocardial ischemia, and (2) elevation of troponin I and/or CKMB for ≤12 hours, with or without heart block. Patients having cardiac arrest assessed by history and electrocardiogram changes were excluded. According to history and clinical assessment, patients having other causes of shock such as hypovolemia, hemorrhage, sepsis, pulmonary embolism, or anaphylaxis were not enrolled. All female patients having shock because of mechanical complications to MI or having severe aorta valve regurgitation/stenosis were also excluded.

Data regarding gender, age, past history of MI, congestive heart failure, angina, hypertension, diabetes, or smoking were noted. In all patients, outcomes (i.e., sinus bradycardia, sinus tachycardia, atrial fibrillation, complete heart block, and death) were recorded during the hospital stay. Sinus bradycardia was defined as regular but unusually slow heart beat (50 beats/minute or less at rest). Sinus tachycardia was defined as elevated rate of impulses, defined as a rate greater than 100 beats/minute in an average adult. Atrial fibrillation was described as absence of P-waves and presence of QRS complexes in an irregular rhythm with or without the presence of coarse or fine fibrillatory waves. Complete heart block was labeled as complete absence of AV conduction, where none of the supraventricular impulses are conducted to the ventricles. Mortality was noted within three days of hospital stay and described as more than five minutes of no signs of life, including no blood pressure, no pulse, no cardiac and respiratory sounds, and pupils bilateral dilated and fixed. All cases were considered for thrombolytic agents if there was no known contraindication. If any complications occurred, they were managed as per the institutional protocols.

All study data were recorded on a predesigned proforma. SPSS version 19.0 (IBM, Armonk, NY, USA) was used for data analysis. Frequencies and percentages were calculated for qualitative variables including diabetes mellitus, hypertension, smoking, obesity, educational status, and outcome. Mean and standard deviation (SD) were calculated for quantitative variables including age and duration of symptoms. Effect modifier including age, diabetes mellitus, hypertension, smoking, obesity, educational status, and duration of symptoms of inferior wall MI were controlled through stratification. Post-stratification chi-square test was applied considering a p-value of ≤0.05 as significant.

## Results

The mean age of study participants was 58.80 ± 9.17 years, while 47 (79.7%) patients were above 50 years of age. The mean onset of duration of signs and symptoms of inferior wall MI was 3.48 ± 1.53 hours. There were 36 (61.0%) patients who had diabetes mellitus, 46 (78.0%) had hypertension, 17 (28.8%) were obese, nine (15.3%) had a family history of MI, and three (5.1%) were smokers. There were 43 (72.9%) patients who were illiterate.

In our study, eight (13.6%) females were found to have sinus bradycardia, seven (11.9%) had sinus tachycardia, three (5.1%) had atrial fibrillation, and 24 (40.7%) had complete heart block. Mortality was noted in five (8.5%) patients.

Stratification with respect to age, duration of onset, diabetes mellitus, hypertension, obesity, family history of MI, smoking status, and educational status was done to observe the effect of these modifiers on in-hospital outcomes (Tables 1, 2, 3). Significant association of tachycardia was noted with obesity (p = 0.009). Atrial fibrillation had significant association with increasing age (p = 0.041). Complete heart block was found to have significant association with increasing age (p = 0.040). Mortality was noted to have significant association with family history of MI (p = 0.004).

**Table 1:** Distribution of sinus bradycardia and sinus tachycardia with respect to study variables. MI, myocardial infarction

Study variables	Sinus bradycardia			Sinus tachycardia		
	Yes (n = 8)	No (n = 51)	P-Value	Yes (n = 7)	No (n = 52)	P-Value
Age						
≤50 years	1 (12.5%)	11 (21.6%)	0.554	2 (28.6%)	10 (19.2%)	0.564
>50 years	7 (87.5%)	40 (78.4%)		5 (71.4%)	42 (80.8%)	
Onset duration						
≤3 hours	3 (37.5%)	26 (51.0%)	0.478	3 (42.9%)	26 (50.0%)	0.722
>3 hours	5 (42.5%)	25 (49.0%)		4 (57.1%)	26 (50.0%)	
Diabetes mellitus	5 (62.5%)	31 (60.8%)	0.926	5 (71.4%)	31 (59.6%)	0.547
Hypertension	7 (87.5%)	39 (76.5%)	0.484	6 (85.7%)	40 (76.9%)	0.598
Obesity	2 (25.0%)	15 (29.4%)	0.798	4 (57.1%)	13 (25.0%)	0.009
Family history of MI	1 (12.5%)	8 (15.7%)	0.934	1 (14.3%)	8 (15.4%)	0.939
Smoker	1 (12.5%)	2 (3.9%)	0.304	0 (0%)	3 (5.8%)	0.514
Illiterate	6 (75.0%)	37 (72.5%)	0.885	5 (71.4%)	38 (73.1%)	0.927

**Table 2:** Distribution of atrial fibrillation and complete heart block with respect to study variables. MI, myocardial infarction

Study variables	Atrial fibrillation			Complete heart block		
	Yes (n = 3)	No (n = 56)	P-Value	Yes (n = 24)	No (n = 35)	P-Value
Age						
≤50 years	2 (33.3%)	10 (17.9%)	0.041	8 (33.3%)	4 (11.4%)	0.04
>50 years	1 (66.7%)	46 (82.1%)		16 (66.7%)	31 (88.6%)	
Onset duration						
≤3 hours	1 (33.3%)	28 (50.0%)	0.574	13 (54.2%)	16 (45.7%)	0.524
>3 hours	2 (66.7%)	28 (50.0%)		11 (45.8%)	19 (54.3%)	
Diabetes mellitus	2 (33.3%)	34 (60.7%)	0.837	14 (58.3%)	22 (62.9%)	0.726
Hypertension	2 (66.7%)	44 (78.6%)	0.628	20 (83.3%)	26 (74.3%)	0.41
Obesity	2 (66.7%)	15 (26.8%)	0.137	11 (45.8%)	6 (17.1%)	0.017
Family history of MI	1 (33.3%)	8 (14.3%)	0.371	3 (12.5%)	6 (17.1%)	0.237
Smoker	0 (0%)	3 (5.4%)	0.386	2 (83.3%)	1 (2.9%)	0.347
Illiterate	2 (66.7%)	41 (73.2%)	0.804	17 (70.8%)	26 (74.3%)	0.77

**Table 3:** Distribution of artial fibrillation and complete heart block with respect to study variables. MI, myocardial infarction

Study variables	Mortality		
	Yes (n = 5)	No (n = 54)	P-Value
Age			
≤50 years	2 (40.0%)	10 (18.5%)	0.254
>50 years	3 (60.0%)	44 (81.5%)	
Onset duration			
≤3 hours	2 (40.0%)	27 (50.0%)	0.669
>3 hours	3 (60.0%)	27 (50.0%)	
Diabetes mellitus	3 (60.0%)	33 (61.1%)	0.961
Hypertension	4 (80.0%)	42 (77.8%)	0.909
Obesity	3 (60.0%)	14 (25.9%)	0.108
Family history of MI	3 (60.0%)	6 (11.1%)	0.004
Smoker	1 (20.0%)	2 (3.7%)	0.113
Illiterate	4 (80.0%)	39 (72.2%)	0.708

## Discussion

Although age-adjusted death rates have been found to be decreasing among developed countries, death rates are rising in Pakistan [14]. Timely detection, hemodynamic support, rapid reperfusion treatment, and ideas about the most probable complications of acute MI can assist in improving the outcome among these patients [15]. Advanced age has been linked with poor short-term prognosis in patients having acute MI [16]. These patients could be less likely to get reperfusion therapy or adjunctive treatment. Gurwtiz and colleagues noted progressive decline in the administration of thrombolytic therapy with advancing age [17]. Clinicians are hesitant to utilize thrombolytic agents in older patients as these patients are found to have delayed presentation, unfamiliar time of symptom onset, increased chances of bleeding, and relative contraindication to thrombolytic agents [18].

After adjusting confounding variables, old age has been marked to be an independent predictor of in-hospital death among patient having acute MI [19]. In the present study, 79.7% female patients were found to be aged above 50 years. Jim et al. noted increasing age to be significantly associated with in-hospital mortality among female patients [20]. Presentation after 24 hours and use of β-blockers have also been linked with mortality [20]. Although this study did not make any comparison in terms of gender, it has been found by previous researchers that female patients below 75 years of age have two-fold increased chances of in-hospital mortality; however, the difference in mortality pattern was not significant after 75 years of age [21]. Recent local data have found that patients having inferior wall acute MI are more prone to have AV nodal conduction issues [22]. Ravikumar et al. noted 60% of the cases where arrhythmias occurred within the first 12 hours of admission [23]. Some researchers have revealed that around 90% of the patients showed cardiac conduction abnormalities within 24 hours following acute MI [24]. In our study, 13.6% females were found to have sinus bradycardia, 11.9% sinus tachycardia, 5.1% atrial fibrillation, and 40.7% had complete heart block. Compared to previous findings, regional data indicate that incidence of conduction blocks has been found to be increasing among patients with inferior wall MI, while AVB and RVI were found to be significantly linked with poor outcomes [25]. Berger and Ryan revealed three high-risk subgroups, namely, complete AV block, precordial ST-segment depression and RVI, among patients having inferior wall MI [7]. Jim et al. found complete AVB to predict in-hospital mortality with an odds ratio of 3.9, whereas its linkage with unfavorable outcomes have been well documented in previous studies [20]. This could be due to the relation of complete AVB with large infarct areas.

In the present study, mortality was noted in 8.5% of the patients which is lower than that found previously (13.4%) in a local study [15]. Short-term mortality rates between 12% and 23% have been noted among patients with acute inferior wall MI [26].

As this was a single center study conducted at an urban healthcare facility with a relatively shorter sample size, the findings cannot be generalized. We also did not have any randomization or control design in the present study. We were also unable to detail management protocols and their linkage with the outcome as this was beyond the scope of our study.

## Conclusions

Women with an acute inferior wall MI had higher rates of complete heart block and adverse in-hospital outcomes. Female gender itself with inferior wall MI could be at risk for short-term in-hospital poor outcomes. Further studies are needed to further confirm the gender differences and appropriate management protocols among patients presenting with inferior wall acute MI.
